# Immunization coverage and factors associated with incomplete vaccination in children aged 12 to 59 months in health structures in Lomé

**DOI:** 10.1186/s13104-019-4115-5

**Published:** 2019-02-14

**Authors:** Wendpouiré I. C. Zida-Compaore, Didier K. Ekouevi, Fifonsi A. Gbeasor-Komlanvi, Essèboè K. Sewu, Tetouyaba Blatome, Adama D. Gbadoe, Diparidè A. Agbèrè, Yawo Atakouma

**Affiliations:** 1 Département de Santé Publique, Faculté des Sciences de la Santé, Université de Lomé, Lomé, Togo; 2 Centre Africain de Recherche en Epidémiologie et en Santé Publique (CARESP), Lomé, Togo; 3 ISPED, Université de Bordeaux & Centre INSERM U1219-Bordeaux Population Health, Bordeaux, France; 4 Département de Pédiatrie, Faculté des Sciences de la Santé, Université de Lomé, Lomé, Togo; 5 Hôpital de Bè, Lomé, Togo; 6 Centre Hospitalier Régional de Lomé-Commune, Lomé, Togo; 7 Centre Hospitalier Universitaire Sylvanus Olympio, Lomé, Togo

**Keywords:** Immunization coverage, Associated factors, Obstacles, Hospital studies, Togo

## Abstract

**Objective:**

To estimate the immunization coverage among children admitted for consultation or hospitalization in health structures of Lomé.

**Results:**

A total of 797 respondent–child couples were included and 31.1% of them had their immunization cards. Complete immunization coverage was 69.3%, 95% confidence interval (65.9–72.5) and per antigen, it ranged from 83.1% for measles to 95.7% for BCG. Factors associated with incomplete immunization were the absence of immunization card (p < 0.001), respondents’ sex (p < 0.001), level of education (p < 0.001), marital status (p < 0.001) and the level of the health structure in the organization of the Togolese health system (p < 0.001). Obstacles to immunization were mainly the lack of money to pay for immunization fees (38.4%) and forgetting the immunization appointment (28.1%).

## Introduction

Immunization is one of the most cost effective health interventions which helps prevent 2–3 million deaths per year [1]. To protect children against vaccine preventable diseases and to facilitate their access to vaccines, the World Health Organization (WHO) and United Nations International Children’s Emergency Fund (UNICEF) have implemented in 1977 the Expanded Program on Immunization (EPI) [2]. In Togo, the implementation of EPI is effective since 1980. Nowadays, it has become one of the country’s public health priorities [3].

Monitoring of immunization program is usually carried out through vaccination coverage surveys. The first surveys conducted in Togo used the WHO cluster survey methodology [4] or household surveys such as the demographic and health surveys (DHS) or the Multiple Indicator Cluster Survey (MICS). According to the third DHS conducted in 2013–2014 in Togo, 61% of children aged 12–23 months were fully immunized based on immunization cards or mother’s recall, and the immunization coverage was higher in urban (66%) than in rural area (59%) [5]. In 2010, immunization coverage was estimated at 63.8% among children aged less than 5 years in the fourth MICS [6].

Vaccination coverage surveys are often difficult to carry out because of their high financial cost, information bias when immunization cards are not available and households characteristics (geographic inaccessibility, climatic and security issues) [7]. Consequently, these surveys are not regularly conducted. However, it is important to monitor vaccination coverage indicators on a regular basis. Other sources of information on immunization coverage, such as hospital studies, must be explored to assess these indicators. Therefore, this study aimed to estimate vaccination coverage among children aged 12–59 months seen in medical consultation or hospitalized in health structures in Lomé.

## Main text

### Methods

#### Study design and population

A cross-sectional study was carried out from April to August 2017 in five health structures in Lomé. Togo health system has a three-level pyramid structure: tertiary, secondary and primary levels. Therefore, we selected at least one health structure at each level of the health pyramid. The study was conducted in the two teaching hospitals (out of two) from tertiary level (Centres Hospitaliers Universitaires Sylvanus Olympio and Campus), two secondary-level health structures (out of two) (Centre Hospitalier Régional Lomé-Commune and Hôpital de Bè) and one randomly selected (out of 140) primary-level health structure (Centre Medico Social Adidogomé).

All children aged 12–59 months hospitalized or seen in consultation in selected health structures during the survey period were eligible for this study. At this age, children should have received all vaccines according to the EPI schedule in Togo. A systematic sampling method was used to select eligible participants. The health card of recruited children was marked with a sticker to avoid double counting.

With a vaccination coverage estimated at 61%, a precision of 4% and a significance level of 5%, the minimum sample size was estimated at 571 respondent–child couples.

#### Data collection

A 10-min pre-tested questionnaire was administered to children’s respondent during a face-to-face interview. Information collected included data on child’s health and immunization, the respondent’s socio-demographic characteristics and knowledge on immunization, as well as the reasons for incomplete immunization. The availability of the immunization card was also assessed. If the card was not available, the vaccine injection sites were showed to the respondent for recall purpose.

#### Definition of variables

Complete immunization coverage was defined as the immunization status of a child who received all the doses of vaccines recommended by the Togo’s EPI, including one dose of tuberculosis vaccine (BCG), four doses of oral poliomyelitis vaccine (OPV), three doses of pentavalent (PENTA) vaccine (conjugate vaccine against diphtheria, tetanus, pertussis, hepatitis viral B and infections to *Haemophilus influenzae b*), three doses of pneumococcus vaccine (PNEUMO), two doses of rotavirus vaccine (ROTA), one dose of measles vaccine, one dose against rubella and one dose against yellow fever [8]. Otherwise, immunization status was defined as incomplete.

A knowledge score on immunization was constructed with five questions based on routine recommendations provided by midwives to parents after childbirth, including the total number of required immunization sessions, number of vaccines to be administered to children, ages at first and last vaccine, and citing at least two EPI vaccines. Each correct answer was worth one point and total score ranged from 0 (no correct answer) to 5 (correct answers to all five items).

#### Statistical analysis

Descriptive statistics were performed and results were presented with frequency tabulations and percentages. Quantitative variables were presented as medians with their interquartile range (IQR). Prevalence rates were estimated with their 95% confidence interval (95% CI). Logistic regression analyses were performed to identify factors associated with “incomplete immunization coverage”. All analyses were performed using R^®^ software.

### Results

A total of 797 were enrolled in the study, resulting in a response rate of 96.1% and 42.9%, 25.5% and 31.6% of study participants were recruited in primary, secondary and tertiary level health structures, respectively.

Respondents were mainly mothers (91.6%), with median age of 30 years, (IQR: 26–34) and 45.5% had a secondary school level education. Children’s median age was 25 months (IQR: 17–36) and 51.7% of recruited children were male (Table 1).

**Table 1:** Sociodemographic characteristics of respondents and children according to the health structure level

Characteristics	Health structures (N = 797)						Total	
	Primary level		Secondary level		Tertiary level			
	n	%	n	%	n	%	N	%
Respondent								
Sex								
Female	321	93.8	197	97.1	238	94.4	756	94.9
Male	21	6.2	6	2.9	14	5.6	41	5.1
Relationship between respondent and child								
Mother	313	91.5	187	92.1	230	91.3	730	91.6
Father	12	3.4	7	3.4	12	4.7	31	3.9
Grand parents	5	1.5	5	2.5	7	2.8	17	2.1
Tutor	2	0.6	1	0.5	0	–	3	0.4
Brother/sister	3	0.9	0	–	0	–	3	0.4
Other	7	2.1	3	1.5	3	1.2	13	1.6
Education level of respondent								
No education	43	12.6	32	15.8	28	11.1	103	12.9
Primary	93	27.2	50	24.6	51	20.2	194	24.3
Secondary	162	47.3	89	43.8	112	44.4	363	45.5
Higher	42	12.3	32	15.8	61	24.3	135	17.0
MD	2	0.6	0	–	0	–	2	0.3
Median age group								
< 30 years	206	60.2	99	48.8	132	52.4	437	54.8
≥ 30 years	128	37.5	103	50.7	120	47.6	351	44.1
MD	8	2.3	1	0.5	0	–	9	1.1
Marital status								
Never in union	48	14.0	21	10.4	14	5.6	83	10.4
Married	262	76.6	182	89.6	230	91.3	674	84.5
Other	32	9.4	0	–	8	3.1	40	5.1
Occupation								
Not working	6	1.7	7	3.4	18	7.1	31	3.9
Salaried employee	21	6.1	13	6.4	45	17.9	79	9.9
Housewife	53	15.5	27	13.3	65	25.8	145	18.2
Retailer (self employed)	122	35.7	79	39.0	47	18.6	248	31.1
Other	140	41.0	77	37.9	77	30.6	294	36.9
Child								
Sex								
Male	157	45.9	115	56.6	140	55.6	412	51.7
Female	185	54.1	88	43.4	112	44.4	385	48.3
Age group (months)								
12–24	149	43.6	120	59.1	129	51.2	398	49.9
24–36	98	28.6	46	22.7	70	27.8	214	26.9
36–59	95	27.8	37	18.2	53	21.0	185	23.2
Place of birth								
Public structure	266	77.8	161	79.3	205	81.3	632	79.3
Private structure	71	20.8	37	18.2	46	18.3	154	19.3
Home	1	0.3	3	1.5	0	–	4	0.5
Other	4	1.1	2	1.0	1	0.4	7	0.9

MD: missing data

#### Possession of immunization card

Among recruited children, 31.1% came to the health care center with an immunization card. The proportion of children having an immunization card was 22.2%, 39.9% and 35.3% in primary, secondary and at tertiary level health structures, respectively (p < 0.001). The reasons for the absence of the immunization card were: leaving the card at home (91.9%), never having a card for the child (4.6%) and the loss of the card (3.5%).

#### Immunization coverage

Complete immunization coverage in our study was 69.3% (95% CI 65.9–72.5). It was 70.0% in primary level, 76.1% in the secondary level and 62.8% in tertiary level health structures (p = 0.005).

Among the 541 children who did not have an immunization card, complete immunization coverage was 62.3% and was 84.6% among the 247 children who had immunization cards (p = 0.002).

For all children, this coverage was 95.7%, 94.7% and 87.6% for BCG, the first dose (OPV-0) and the fourth dose of poliovirus vaccine (OPV-3), respectively. For pentavalent vaccine, the coverage ranged from 94.2% for PENTA 1 to 87.8% for PENTA 3 while it was 83.1% for measles and 71.9% for yellow fever (Table 2).

**Table 2:** Immunization coverage of children by antigen

	Health structures (N = 797)									Total		
	Primary level			Secondary level			Tertiary level					
	N	n	%	N	n	%	N	n	%	N	n	%
*All children*	342			203			252			797		
BCG		330	96.5		190	93.6		243	96.4		763	95.7
PENTA 1/PNEUMO 1		329	96.2		190	93.6		213	84.5		751	94.2
PENTA 2/PNEUMO 2		326	95.3		188	92.6		222	88.1		736	92.4
PENTA 3/PNEUMO 3		306	89.5		178	87.7		216	85.7		700	87.8
OPV-0		328	95.9		185	91.1		242	96.0		755	94.7
OPV-1		327	95.6		186	91.6		231	91.7		744	93.4
OPV-2		325	95.0		184	90.6		222	88.1		731	91.7
OPV-3		307	89.8		177	87.2		214	84.9		698	87.6
ROTA-1		289	84.5		171	84.2		197	78.2		657	82.4
ROTA-2		291	85.1		166	81.8		198	78.6		655	82.2
Measles/rubella		245	71.6		156	76.8		178	70.6		579	83.1
Yellow fever		241	70.5		159	78.3		173	68.6		573	71.9

BCG: Bacillus Calmette–Guerin; Penta: pentavalent vaccine (vaccine against diphtheria, Tetanus, pertussis, hepatitis B and Haemophilus influenzae); OPV: oral polio vaccine; Pneumo: Pneumococcus conjugated vaccine; Rota: rotavirus vaccine

#### Knowledge on immunization

Nearly 62.9% of respondents were unaware of the number of required immunization sessions and 96.0% did not know the number of vaccines children must receive. Almost three-quarters (73.2%) of respondents had cited two Togo EPI vaccines. Ages at first and last vaccine were given by 4.7% and 20.3% of respondents, respectively. Knowledge scores on immunization varied from 0 (7.9%) to 3 (8.9%) and a score of 1 and 2 was reported for 45.0% and 38.2% of respondents respectively. Respondent’s knowledge on immunization was higher in primary level health structures (75.0% with a score of 3/5) compared with that of respondents of tertiary level health structures (25.0% with a score of 3/5) (p = 0.076).

#### Barriers to immunization

Among respondents of children with incomplete immunization (n = 242), 38.4% did not have money to pay for immunization fees; 28.1% reported that they forgot the immunization appointment and 8.7% did not have time to take their children to a health care center for vaccination.

The long waiting time at the health structure (5.4%), the lack of vaccines (4.1%) and the long distance from home to the health structure (2.1%) were cited as the main contextual factors. Cultural beliefs or prohibitions were mentioned by 11.6% of respondents of children who were partially immunized.

#### Factors associated with incomplete immunization

After adjustment on the other variables, being a male respondent (aOR: 2.7; 1.3–5.7; p < 0.001), being not married (aOR: 2.6; 1.6–3.9; p < 0.001), having primary education level (aOR: 2.2; 1.6–3.2; p < 0.001), not having an immunization card (aOR: 3.5; 2.4–5.4; p < 0.001) and attending primary level health structure (aOR: 2.0; 1.3–3.2; p < 0.001) were associated with incomplete immunization (Table 3).

**Table 3:** Factors associated with incomplete immunization coverage

Characteristics of respondents (N)	Children fully immunized		Children partially or no immunized		Univariate analysis			Multivariate analysis		
	N	%	N	%	OR	95% CI	p-value	aOR	95% CI	p-value
Sex (n = 785)							0.008			< 0.001
Female	527	96.9	223	92.5	1			1		
Male	17	3.1	18	7.5	2.5	[1.3; 4.9]		2.7	[1.3; 5.8]	
Relationship between respondent and child (n = 784)							0.002			
Mother	515	94.5	210	87.9	1					
Other	30	5.5	29	12.1	2.4	[1.4; 4.1]				
Education level (n = 787)							< 0.001			< 0.001
> Primary	371	67.9	123	51.0	1			1		
≤ Primary	175	32.1	118	49.0	2.0	[1.5; 2.8]		2.2	[1.6; 3.1]	
Median age group (n = 780) (years)							0.323			
≥ 30	247	45.7	100	41.8	1					
< 30	294	54.3	139	58.2	1.2	[0.9; 1.6]				
Marital status (n = 787)							< 0.001			< 0.001
Married	484	88.6	184	76.4	1			1		
Other	62	11.4	57	23.6	2.4	[1.6; 3.6]		2.6	[1.6; 4.0]	
Religion (n = 786)							0.0022			
Catholic	232	42.6	75	31.1	1					
Protestant	155	28.4	66	27.4	1.3	[0.9; 1.9]				
Muslim	72	13.2	52	21.6	2.2	[1.4; 2.5]				
Other	86	15.8	48	19.9	1.7	[1.1; 2.7]				
Occupation (n = 785)							0.002			0.053
Not working	23	4.2	7	2.9	1			1		
Salaried employee	66	12.1	12	5.0	0.6	[0.2; 1.8]		0.6	[0.2; 1.9]	
Housewife	81	14.9	63	26.3	2.6	[1.1; 6.8]		1.8	[0.7; 5.1]	
Retailer (self employed)	174	31.9	71	29.6	1.3	[0.6; 3.5]		1.1	[0.4; 3.1]	
Other	201	36.9	87	36.2	1.4	[0.6; 3.3]		1.2	[0.5; 3.2]	
House type (n = 786)							0.3944			
Multiple dwelling unit	323	59.3	135	56.0	1					
Single family home	222	40.7	106	44.0	1.1	[0.8; 1.6]				
Health structures (n = 788)							0.008			<0.001
Secondary level	153	28.0	48	19.8	1			1		
Tertiary level	157	28.8	93	38.5	1.4	[0.9; 2.0]		1.0	[0.7; 1.6]	
Primary level	236	43.2	101	41.7	1.9	[1.2; 2.9]		2.0	[1.3; 3.2]	
Sex of the child (n = 786)							0.3544			
Female	266	48.8	109	45.2	1					
Male	279	51.2	132	54.8	1.1	[0.8; 1.6]				
Age of child (n = 788) (months)							0.9514			
12–24	286	52.4	124	51.2	1					
24–36	136	24.9	61	25.2	1.0	[0.7; 1.5]				
36–59	124	22.7	57	23.6	1.1	[0.7; 1.5]				
Birth order (n = 764)							0.031			
1	213	40.0	70	30.1	1					
2	162	30.5	80	34.5	1.5	[1.0; 2.2]				
≥ 3	157	29.5	82	35.4	1.6	[1.1; 2.3]				
Availability of immunization card (n = 779)							< 0.001			< 0.001
Yes	209	38.7	38	15.9	1			1		
No	331	61.3	201	84.1	3.3	[2.3; 4.9]		3.5	[2.4; 5.4]	
Knowledge level (n = 89)							0.197			
≥ 2	32	46.4	6	30.0	1					
≤ 1	37	53.6	14	70.0	2.0	[0.7; 6.3]				

aOR: adjusted odds-ratio; CI: confidence interval

### Discussion

Overall, complete immunization coverage observed in the present study is similar to that reported in household surveys in 2013 and 2017 in Lomé with 62.2% and 72.3% respectively [6, 9]. Although comparisons of immunization coverages must be done with caution because of difference in studies’ methods (including the availability of immunization cards and the age range of study population), it should be noted that carrying out hospital-based study in Togo could be an opportunity to obtain immunization indicators at no additional cost. Indeed, in Togo, data on maternal and infant deaths are compiled weekly and immunization data can be collected as part of this existing surveillance system.

The immunization card is a paper used to record and track immunization coverage. In Togo, for household surveys conducted in 2012 and in 2013, it was available for 77% and 70% of recruited children, respectively [5, 10]. Difference in the availability of immunization card can be explained by the study setting and the study population. The proportion of children who came to the hospital with their immunization card was greater than that of 24% observed in a multicenter study conducted in Cameroon, Central African Republic and Senegal, among hospitalized children aged 3 months to 6 years between April 2009 and May 2010 [11]. In our study, nine respondents in ten who did not have the immunization card declared that the card was left at home while the children of household surveys were aged 12–23 months, with higher odds of immunization card retention [12].

Not having an immunization card was associated with incomplete immunization. Similar findings about immunization card have been reported in Senegal (aOR: 8.27; 95% CI 4.18–16.50) and in Ghana (aOR: 50.30; 95% CI 14.40–175.92) [13, 14]. Using web based tracking system and/or mobile phone could be innovative ways to monitor the immunization coverage of children and reduce the risk of losing the card. However, studies should be carried out to assess these interventions’ efficacy and sustainability. Currently, in Côte d’Ivoire, children can be registered in a database at the first visit for BCG to allow for catch up of vaccines, but this approach has not been yet evaluated. In their studies, Abdulraheem et al. and Wiysonge et al. [15, 16] reported that the lack of time, the poor education level of mother and the poor economic household were strongly associated with incomplete immunization. A study conducted in six West African countries found that being born at home, mothers lacking access to the media, family poverty and illiteracy of mothers were factors associated with incomplete immunization [17]. Health authorities should organize immunization campaigns to allow for catch-up of missed vaccines. Also, there is a need to strengthen sensitization on immunization since the knowledge score of respondents is low.

Barriers to incomplete immunization in our study were also reported by Ndiaye et al. [13] in Senegal, Makoutodé et al. [18] in Benin. In Cameroon and Togo, studies mentioned financial and geographic inaccessibility as main bottlenecks for complete immunization [11, 19]. Immunization costs have been reported in this study; the respondents declared that they have to pay a certain amount for consumables or immunization card during the EPI sessions which should be free.

This hospital survey was easier to carry out in terms of time and costs than household surveys. It must lead to change in routine practices of health providers such as the systematic verification of immunization cards at each consultation or hospitalization and the application of catch-up strategies for missed vaccines.

## Limitations

Our study has some limits: PENTA and PNEUMO vaccines are administered during the same immunization session but in some immunization cards, only the date of immunization was reported; vaccines names and tags were not recorded. Therefore, we could not determine with precision which vaccine was administered, leading to an underestimation of antigen specific immunization coverage among children who had their immunization card. Moreover, the study was restricted to Lomé and the results cannot be extrapolated nationwide. In this survey, for the children who have not an immunization card, the immunization status was determined based on declarative data of respondents; this could be a source of memory and classification bias with an underestimation or overestimation of children’s immunization status.

## Abbreviations

95% CI: 95% confidence interval
aOR: adjusted odds ratio
BCG: tuberculosis vaccine
EPI: Expanded Program on Immunization
IQI: interquartile interval
MD: missing data
MICS4: Fourth Multiple Indicator Cluster Survey
OPV: oral polio vaccine
PENTA: pentavalent vaccine
PNEUMO: pneumococcal vaccine
ROTA: vaccine against rotavirus gastroenteritis
UNICEF: United Nations International Children’s Emergency Fund
WHO: World Health Organization
